# Changing life expectancy in Europe 1990-2019: Global Burden of Disease Study 2019: Nicholas Steel

**DOI:** 10.1093/eurpub/ckac130.197

**Published:** 2022-10-25

**Authors:** N Steel, C BauerStaeb, J Ford, T Gillam, J Schmidt, AS Hughes

**Affiliations:** 1 University of East Anglia, Norwich Medical School, Norwich, UK; 2 Office for Health Improvement and Disparaties, Department of Health and Social Care, London, UK; 3 Primary Care Unit, University of Cambridge, Cambridge, UK

## Abstract

Improvements in life expectancy have slowed in high income countries, with uncertain causes. We assessed the contribution of different causes of death to changes in life expectancy, and changes in population exposure to major risk factors in 16 European Economic Area countries plus the 4 nations of the United Kingdom from 1990-2013 and 2013-2019, using the Global Burden of Disease Study. After decades of steady improvements in life expectancy, all countries experienced smaller annual improvements after 2013. Norway experienced the smallest mean annual rate of change in improvement from pre 2013 to post 2013 of 0.03 years, and Northern Ireland (followed closely by Scotland and England) experienced the largest annual reduction from pre to post 2013 of 0.25 years. The cause of death responsible for the largest reductions in life expectancy improvements after 2013 was cardiovascular disease, followed by neoplasms. The largest reductions in deaths from cardiovascular disease were attributable to seven major risk factors: high LDL cholesterol, tobacco, dietary risks, high fasting plasma glucose, high systolic blood pressure, high body mass index, and low physical activity. The risk factors for deaths from neoplasm were similar. Exposure to tobacco remains a high risk but exposure declined steadily. Exposure to the other risks generally worsened after 2013, particularly for BMI and high fasting plasma glucose. The European countries that had better maintained reductions in deaths from cardiovascular disease and neoplasms also experienced larger improvements in life expectancy. These changes were underpinned by changing exposure to major risks. Policy responses to the slowdown in life expectancy improvements should include reducing population exposure to major risks, including the broader risks from diet and low physical activity, through prevention and addressing the broad social and commercial determinants of health as well as adequate funding for health services.

**Key messages:**

- Reductions in European life expectancy improvements have been driven by changes in deaths from cardiovascular disease and neoplasms.
- Policy responses to the slowdown in life expectancy improvements should include reducing population exposure to major risks, including the broader risks from diet and low physical activity.

